# Psychosocial and Quality of Life Outcomes of Prosthetic Auricular Rehabilitation with CAD/CAM Technology

**DOI:** 10.1155/2014/393571

**Published:** 2014-03-31

**Authors:** Chi Keung Tam, Colman Patrick McGrath, Samuel Mun Yin Ho, Edmond Ho Nang Pow, Henry Wai Kuen Luk, Lim Kwong Cheung

**Affiliations:** ^1^Oral and Maxillofacial Surgery, Faculty of Dentistry, The University of Hong Kong, Hong Kong; ^2^Dental Public Health, Faculty of Dentistry, The University of Hong Kong, Hong Kong; ^3^Department of Applied Social Studies, City University of Hong Kong, Hong Kong; ^4^Oral Rehabilitation, Faculty of Dentistry, The University of Hong Kong, Hong Kong; ^5^Dental Technology, Faculty of Dentistry, The University of Hong Kong, Hong Kong

## Abstract

*Introduction*. The psychosocial and quality of life (QoL) of patients with deformed or missing ears are frequently compromised. The aim of this study is to develop innovative techniques using CAD/CAM technology in prosthetic auricular rehabilitation and provide improvement in the treatment outcomes, including their psychology and QoL. *Methods*. This is a preliminary clinical cohort study. Six patients requesting for auricular reconstruction were recruited and rehabilitated with implant-supported prosthesis using CAD/CAM technology. Different treatment outcomes including QoL and psychological changes were assessed at different time points. *Results*. A significant reduction in severity of depressive symptoms (*P* = 0.038) and an improving trend of satisfaction with life were found at 1 year postoperatively when compared with the preoperative findings. The domain scores in ‘‘Body image”, ‘‘Family/friends/strangers”, and ‘‘Mood” were also significantly higher (P < 0.05) at 1 year postoperatively than 1 week postoperatively. However, only 50% of the patients wear their auricular prosthesis regularly. *Conclusion*. This preliminary study has confirmed that implant-supported auricular prosthesis could induce improvement in the psychology and QoL with statistically significant differences in the domains of the body image, social interaction, and mood. Our present findings can inform research design and hypotheses generation of future studies.

## 1. Introduction

One option to consider in auricular reconstruction, particularly when there has been traumatic loss, is the application of a manufactured auricular prosthesis. Thorne et al. [1] offered a good overview of indications, technique, and difficulties with the use of auricular prostheses. Prosthetic restoration minimizes or eliminates the need for surgical procedures. The prosthetic ear can be retained using mechanical retention such as undercuts or conformers. It can also be attached directly to the skin by using different types of adhesives such as skin bonding cements, two-sided tapes and water soluble, or silicone based adhesive [2]. However, this technique was not well tolerated due to inconvenience, difficulty with retention due to the ineffectiveness of chemical adhesives, skin inflammation, and corrosion of the prosthesis due to its chemical content [3]. Since the international release of osseointegration biotechnology in 1982, it created a large impact on extraoral craniofacial reconstruction, with increasing reports confirming the efficacy of this modality for rehabilitation. Facial prosthetic reconstruction had, by then, entered a new era and became a viable option in selected patients [4–6]. With the advent in such modality of treatment, the problems of prosthetic retention and inconvenience were reduced [7–10].

Technological advancement has also refined prosthesis manufacturing processes [11]. The use of CT data with digital model reconstruction provides the freedom and accuracy for surgeons in planning, manipulating, designing, and fabricating prosthetic ears on a computer screen. The direct digital prosthetic auricular fabrication method utilizes a three-dimensional (3D) image processing technique to determine the form and position of the digital ear model. The digital model on screen can then be used to form a silicone ear either indirectly through making of a mould or directly by forming a wax pattern using rapid prototyping (RP) technology. This makes an immediate prosthesis insertion possible.

It has long been recognized that facial visual difference has the potential for psychological and social detriment on patients with craniofacial defects and that craniofacial rehabilitation has a tremendous psychosocial impact on these patients [12–14]. Unfortunately, psychological assessment and treatment outcome evaluation are often overlooked, which should be integrated into the overall treatment. While there is a developing interest to measure patient response to craniofacial osseointegration treatment, there is currently very little scientific data on the subject. Quality of life (QoL) in facial rehabilitation has largely not been researched. Prospective studies that quantify QoL related to surgical measures are also lacking.

We hypothesize that patients with missing or deformed ears will have positive psychological and QoL changes after reconstruction with CAD/CAM auricular prosthesis. The aims of this clinical series were to evaluate the treatment outcomes of prosthetic auricular rehabilitation, including QoL and clinical psychological changes, implant success, accuracy of implant positioning, morbidities, retentiveness, and appearance of ear prostheses.

## 2. Materials and Methods

The ethical approval for this clinical study was granted by the Institutional Review Board of the University of Hong Kong/Hospital Authority Hong Kong West Cluster (HKU/HA HKW IRB): UW 10-081.

### 2.1. Study Design

This is a prospective clinical cohort study.

### 2.2. Patient Selection

Patients seeking auricular reconstruction were recruited from the Prince Philip Dental Hospital and Queen Mary Hospital from 2010 to 2013. The defects presenting might be due to tumour resection, traumatic injuries, burn, or congenital conditions.

The inclusion criteria were missing or severely deformed ears requesting for auricular reconstruction, age 16 or above and had achieved radial bone epiphyseal closure, failed or refused autogenous ear reconstruction, committed to maintain the prosthesis, and attend follow-up appointments.

The exclusion criteria were radiotherapy given prior to implant surgery, concomitant medical treatments and conditions that affected bone healing, alcohol or drug abuse, psychiatric diseases, pregnancy, and inability to attend follow-up according to schedule.

Patients who fulfilled the above criteria were informed about the research details during the consultation. Each patient was also informed regarding the risks and treatment options. Patients who were willing to participate in the study and attend follow-up appointments were asked to sign an informed consent and proceed with the preoperative evaluation and preparation.

### 2.3. Preoperative Preparation

A multidisciplinary team consisting of maxillofacial surgeons, engineers, prosthodontists, and maxillofacial technicians were involved in the planning stage. All patients underwent conjoint surgical prosthodontic assessment and planning. A preoperative protocol was completed at least one week before the operation. Data collected included the following: age, diagnosis, relevant medical history, previous reconstruction, and associated craniofacial defects. Patients also completed a set of questionnaires for the evaluation of presurgical psychological profile and QoL. Plain photographs, 3D photos using* 3dMDFace* (3dMD, Atlanta, USA), plain radiographs, and spiral CT scan with a* HiSpeed/Fxi* scanner (General Electric, Milwaukee, USA) were also recorded. The CT scan imaging data set was used for the planning, simulation of implant insertion, and visualization of optimal prosthetic positioning.

### 2.4. Digital Biomodel Reconstruction

The CT scan DICOM data set of each patient was converted to STL format using* Mimics* software (v11.11, Materialise, Leuven, Belgium). With the derived STL file,* Fused Deposition Modeling* (FDM) technology was used to generate a biomodel.

### 2.5. Fabrication of the Auricular Prosthesis

The auricular prosthesis was prefabricated individually using the software* Mimics* (v11.11),* Magics* (v13), and* RSM* (v4.0) (Materialise, Leuven, Belgium). A mirroring technique was used to copy the normal contralateral ear morphology along the plane of symmetry. A wax duplicate of the mirrored image was made which is supported by a piece of acrylic substructure on which two housings were made to receive the implant magnet attachments.

The wax ear was then converted to a silicone ear using the conventional techniques. Adhesion between the silicone and the acrylic substructure was achieved using a thin layer of primer.

During the pick-up stage, magnet attachments were positioned onto the implant abutments; cold-cured acrylic resin was mixed and applied to the housings of the acrylic substructure and to the mechanical retentive part of the magnet attachments. The silicone ear was then positioned and the acrylic resin was allowed to set. The magnet attachments were then mechanically locked to the acrylic substructure of the silicone ear.

Functional movements were checked when constructing the second/final prosthesis. For immediate placement, no functional movement checking was applicable as soft tissue changes were anticipated during the first few months after implant installation.

### 2.6. Surgical Technique

The insertion of the implants was either done under local anaesthesia with intravenous sedation or under general anaesthesia. During the operation, the implant drilling was guided by the surgical navigation system (BrainLAB, Feldkirchen, Germany). The calibrated handpiece was used to transfer the virtual plan to the real patient. A reference star-array was secured directly onto the skull bone or with a headband. Trackers were rigidly fixed onto the surgical instruments for calibration and integrated to the navigation system. Two* Ankylos* titanium dental implants (Friadent, Dentsply, Mannheim, Germany) of appropriate length and diameter were inserted according to the planned trajectories. The implants were immediately connected to a prosthetic abutment that had a magnetic attachment. The prefabricated prosthetic ear was then lined with cold-cured acrylic resin and connected in the planned position.

### 2.7. Postoperative Management

Immediately after surgery and during recovery, adhesive tapes and a pressure dressing were used to provide pressure for postoperative haemostasis and reduction of oedema. An experienced nurse was responsible for general monitoring and local wound care. Instructions such as home care, not to sleep on the operated side, and routine hygiene of implant sites as well as the daily prosthesis usage pattern and removal were provided before the patients were discharged.

### 2.8. Outcome Variables

The primary outcome of the study was the changes in quality of life. This was assessed by using standardized questionnaires within 1 month before the surgery and then postoperatively at different treatment time points. The secondary outcomes of the study were implant success, accuracy of implant positioning, morbidities, retentiveness and appearance of ear prostheses, and clinical psychological changes.

### 2.9. Assessment of Psychological Profile and QoL

Each patient was asked to complete a package of self-explanatory questionnaires within 1 month before the surgery (Time 1) and repeated again at 1 week (Time 2), 3 (Time 3) and 6 months (Time 4), and 1 (Time 5) and 2 years (Time 6) after the operation.

The series of questionnaires used for the assessment of psychological profile were as follows.

(1) Hope Scale (HS) [15, 16]; (2) Life Orientation Test-Revised (LOT-R) [17]; (3) Attention to Positive and Negative Information (APNI) Scale [18, 19] Short Form; (4) Hospital Anxiety and Depression Scale (HADS) [20]; (5) Satisfaction with Life Scale (SWLS) [21–23]; (6) Social Avoidance and Distress (SAD) Scale [24, 25]; (7) Posttraumatic Growth Inventory [26–30].

The series of questionnaires used for the assessment of quality of life profile were as follows.

(1) Generic health-related QoL: SF-36 [31–33]; (2) Condition specific health-related QoL: the Toronto Outcome Measure for Craniofacial Prosthetics (TOMCP) [34, 35]. The Chinese version of the instrument, subjected to validation, consists of a 52-item measure with ten domains/subscales.

### 2.10. Assessment of Clinical Outcomes

Clinical morbidities such as morbidity to adjacent vital structures, infection, bleeding, soft tissue redness, tenderness, granulation tissue formation, tissue overgrowth, difficulty of cleaning the implant abutment, and loosening of the abutment were collected using standardized questionnaires intraoperatively and postoperatively at regular intervals of 1 and 2 weeks, 3 and 6 months, and 1, 1.5, and 2 years. Implant success, survival, and failure were evaluated according to the criteria of the International Congress of Implantologist (ICOI) Pisa Consensus [36]. The retentiveness and appearance of the ear prosthesis were assessed using standardized questionnaires. Data collected include the following: marginal accuracy, retention, stability, function, symmetry/position, texture, color stability, and patient acceptance.

### 2.11. Statistical Analysis

IBM,* SPSS* statistics 19.0.0 (US) was utilized for data analysis. Descriptive statistics was applied for demographic data. Changes in the patient's depression symptoms, anxiety, and satisfaction with life were analyzed using Friedman test. For the QoL, descriptive statistics (mean, median, and mode) were calculated for each outcome variable (PCS, MCS, TOMCP, and its domains) at each time point. Linear mixed model analyses were performed to determine if there were significant changes in the scores of each outcome variable over the different time periods. For SF-36 (PCS and MCS) changes from preoperative status were used as a reference, that is, health status prior to prosthetic placement. For TOMCP, since its items are concerned with prosthesis changes from its first usage, postoperative review (1 week) was used as a reference, that is, condition specific QoL following prosthetic placement. Comparisons were made at each postoperative time point compared to the reference time-point (preoperative period for SF-36 and 1 week postoperative for TOMCP) using paired *t*-tests for comparison of mean values. The level of significance (*P* value) was set at 0.05.

The present project focuses on multiple cases study (*n* = 6) among a rare group of patients. We understand that our sample size may not achieve the desirable power of analysis to detect statistical significance, and some of our nonsignificant results may be due to *β*-error (false negative). To check whether the nonsignificant results were due to a lack of statistical power, a post hoc power analysis was conducted using G*Power 3 [37, 38] with power set at the recommended level of 0.80 [37] and *α* = 0.05. It revealed that the sample size would have to increase up to 34 to reach the desirable power. Larger sample size could be recruited in future to examine if our present results can be replicated.

## 3. Results

A total of 6 patients had implants inserted to support an immediately connected auricular prosthesis. There were 4 males (66%) and 2 females (33%). The mean age of the patients was 26.27 years (ranged from 19 to 35 years). Half the patients had completed a 2-year review. Four patients were diagnosed with hemifacial microsomia (HFM) (2 right side and 2 left side). One patient also had cleft palate. The other two patients had microtia (1 right and 1 left).

**Figure 1:**
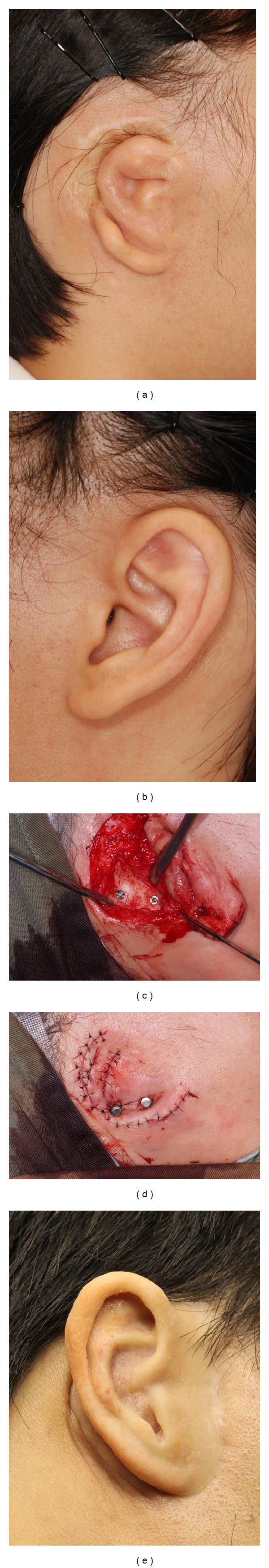
Case illustration of a patient with microtia. (a) Patient's right ear showing microtia. (b) Patient's left ear showing normal shaped ear. (c) Two implants were placed into the mastoid bone. (d) Wound closure with healing abutment exposed. (e) Patient's right ear showing the auricular prosthesis.

The surgical sequence involved in the auricular reconstruction in one of the cases is presented in Figure 1. Four out of six patients (66%) had undergone multiple reconstructive surgeries previously: 3 had distraction osteogenesis; 1 had cleft palate repair; 2 had multiple surgical procedures: scapular osseomyocutaneous flap reconstruction in one patient; in another: multistaged autogenous auricular reconstruction including costochondral cartilage graft, ear lobe revision, and cutaneous graft from the hip. Associated craniofacial defects were particularly prominent in patients who suffered from HFM even though reconstruction had been carried out. There were hypoplasia in their maxilla, mandible, soft tissue, and slanting pupillary level downward of the affected side. None of the 6 patients had previous history of prosthesis use.

**Figure 2:**
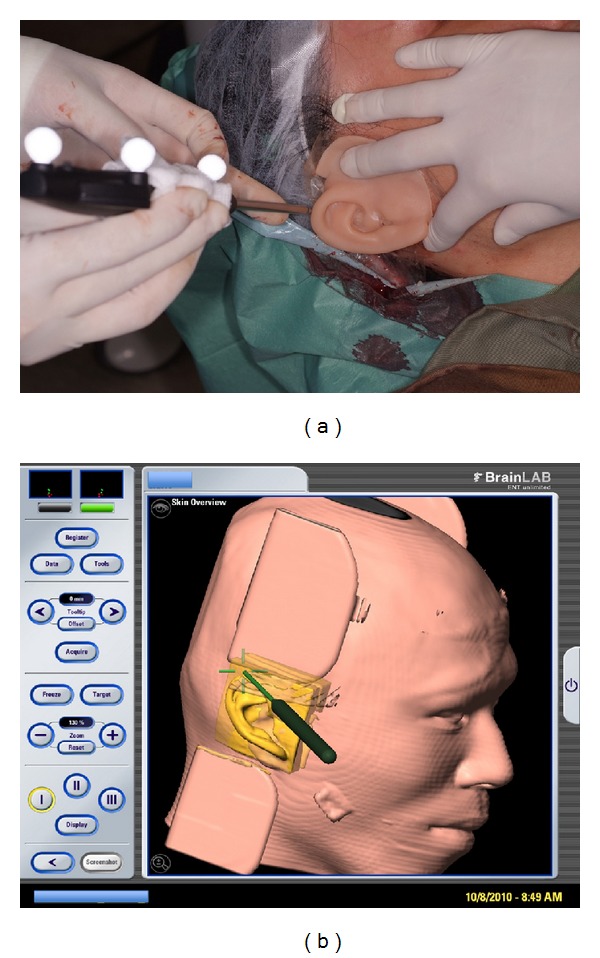
(a) Prosthesis position checked with* Soft Touch* (BrainLAB) digitized probe. (b) Screen shot of checking of the prosthesis position using the* Soft Touch* digitized probe to compare with preplanned position intraoperatively.

The complications encountered during the connection of the implants as well as the insertion of the ear prosthesis are presented in Table 1. The position of immediately connected prosthesis was checked intraoperatively using navigation with* Soft Touch* (BrainLAB, Feldkirchen, Germany) digitized probe on screen to compare with the preplanned position (Figure 2). The result showed the following: good marginal accuracy—4 out of 6 cases, good retention—4 out of 6 cases, good stability—6 out of 6 cases, adequate function—3 out of 3 cases, and symmetrical/good position—6 out of 6 cases.

### 3.1. QoL Outcomes

Questionnaires for assessing patients' QoL changes were administered at the same time points as those for psychological assessment. Accordingly, 6 patients completed both the SF-36 and the TOMCP at Time 1 (pre-op) and 3 of 6 patients were included for longitudinal analyses up to 1 year (Time 5). Time 6 (2 years) was excluded.

### 3.2. Changes in Generic Health-Related QoL

There were significant changes in SF-36 physical-health component scores (PCS) over the study period (*P* = 0.003), (Table 2). At 1 week postoperative there was a significant reduction in SF-36 scores (deterioration in QoL) compared to preoperative status, *P* < 0.01. At all other postoperative time points there were no statistical significant difference in PCS scores compared to pretreatment *P* > 0.05. There were no significant changes in SF-36 mental-health component scores (MCS) over the study period (*P* > 0.05), (Table 3). Furthermore at no postoperative time point were MCS values significantly different from preoperative MCS, *P* > 0.05.

### 3.3. Changes in Condition-Specific QoL

The summary (overall) TOMCP scores increased over time. However, there were no significant changes in TOMCP scores over the study period (*P* = 0.121). At the 1-year postoperative review TOMCP scores were significantly different from 1-week postoperative scores (1 week), *P* < 0.05 (Table 4). Of the ten TOMCP domains, there were significant changes in three of them over time (Table 5).

### 3.4. Implant Success

A total of 12 implants were inserted in patients. According to the criteria of the International Congress of Implantologist (ICOI) Pisa Consensus [36], 10 of the 12 implants were assessed to be successful in attaining good stability and function. Two implants, which were inserted in Case 2, are still submerged. The abutments, which were custom-made, were removed due to persistent adverse periabutment skin response.

### 3.5. Implant Abutment

At 6-month postoperative assessment, there were 3 patients who refused to wear the auricular prosthesis since the last review (Postoperative 3-month). The reasons were (i) nonretentive prosthesis (3 patients) and (ii) colour mismatch (1 patient). The problems encountered by these 3 patients were not addressed as they did not return for follow-up.

The mean exposures of the abutment are listed in Table 6. There was a difference in the clinical implant abutment exposure between patients willing and those unwilling to wear the prosthesis. In patients who refused to wear the prosthesis, a significant decrease (*P* < 0.05) in the superior implant abutment exposure was noted in 1-2 weeks postoperative and 6 months postoperative. Each patient showed at least one implant abutment almost submerged beneath the skin. This was believed to be the cause for the poor retention as complained by the patients who were unwilling to wear the prosthesis. On the other hand, an increase in abutment exposure was generally observed in patients willing to wear the prosthesis in 1-2 weeks postoperative and 6 months postoperative (Table 7).

Discoloration was also noted in 2 out of 3 patients who were willing to wear the prosthesis. In one patient the discoloration started since 3 months postoperative and he needed to compensate it by applying cosmetic make-up onto the prosthesis. In another patient the discoloration became evident after 18 months posttreatment. All implants were considered to be without any signs of infection. There was no implant abutment loss due to trauma.

**Figure 3:**
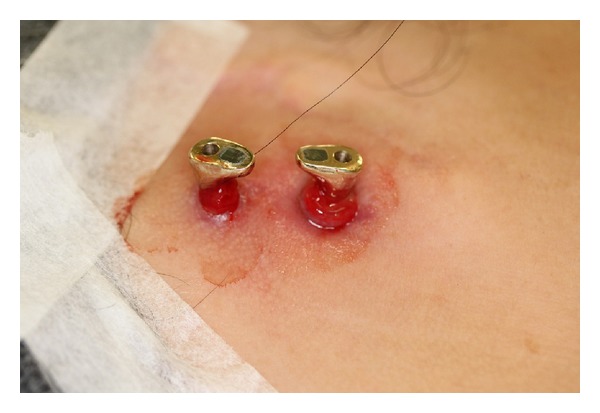
Persistent adverse periabutment skin response around the gold alloy abutments.

In 3 cases, magnet attachments were used. Two cases had ball with snap attachments. One patient (Case 2) had 2* Ankylos* straight standard abutments (Friadent, Dentsply, Mannheim, Germany) found loosened postoperatively. They were later replaced with custom-made gold abutments. However, these 2 gold abutments had to be removed due to persistent soft tissue inflammation (Figure 3) which was controlled after placement with sulcus formers for a prolonged period.

### 3.6. Skin/Soft Tissue

After fitting with the ear prosthesis, no bleeding on probing was found around the implants [39]. There was also no implant threads exposure. Redness/moist tissue, infection, tenderness, granulation tissue, and tissue overgrowth were not observed.

### 3.7. Peri-Implant Soft Tissue (Holgers Scale)

For the first 3 postoperative periods (1 week, 2 weeks, and 1 month) assessed, Holgers scale was not applicable as the wound healing process had not completed and the soft tissue conditions had not yet consolidated. The length of abutment visible (mm) was observed to be closely related to the skin/soft tissue and scores on the Holgers Scale. The greater the length of the abutment was exposed above the skin, the better the clinical outcome. Skin thinning had been performed in all patients. This was performed to reduce the thickness of the subcutaneous tissue and temporalis muscle. However, after a period, periabutment soft tissue thickness increased again in Case 2.

### 3.8. Psychological Outcome

At Time 1 (pre-op), 6 patients completed the psychological assessment on hope, attentional bias, and optimism. Hope was measured with an Adult Trait Hope Scale. The short form Chinese version Attention to Positive and Negative Information Scale (APNIS) measured attentional bias. Life Orientation Test (LOT) measured optimism. Only 3 of 6 patients provided sufficient data for longitudinal analyses up to 1 year (Time 5). Time 6 was excluded from further analysis due to insufficient data of 2 years. Nonparametric test was employed because of the small number of participants. Friedman test was used to explore changes in depression symptoms and anxiety symptoms from Time 1 (pre-op) to Time 5 (1 year). The results showed a significant decrease in severity of depressive symptoms (*χ*²(4) = 10.15, *P* = 0.038). However, there was no significant change in severity of anxiety symptoms (*χ*²(4) = 5.50, *P* = 0.240). Friedman test was again used to examine changes in Satisfaction with Life from Time 1 (pre-op) to Time 5 (12 months). It was shown that there was an improving trend of satisfaction with life although the statistical result is marginally nonsignificant (*χ*²(3) = 6.52, *P* = 0.089).

## 4. Discussion

The rehabilitation of a missing or deformed external ear can be achieved by using a prosthesis anchored by implants integrated in the skull bone [7]. The use of implant-retained prosthesis is now a well-recognized method for creating an aesthetically acceptable result in auricular reconstruction.

Computer-assisted planning has been recommended for this procedure so that the rehabilitation team can preoperatively plan the implant position and the region of the ear prosthesis. The utilization of surgical navigation has been made to improve intraoperative safety and avoid the damage to critical anatomic structures.

### 4.1. QoL

Over the last few years, QoL assessments have become important in evaluating the quality of medical interventions [40]. There were only 8 papers addressing the QoL of facial prosthesis, with only 2 focusing on auricular prosthesis that both were retrospective studies [40–47]. In contrast, this is the first prospective study on QoL on auricular prosthetic rehabilitation in which we obtained preoperative QoL findings that were important in evaluating changes with the postreconstruction results. Although the QoL assessment of this study was based on a small sample, as a preliminary study, the repeated assessments offer a useful insight into the trajectory of QoL during the first year following prosthetic placement that can inform future research for evidence based practice. Increasingly, it is recognized that patients' perceptions are important in assessing health needs and in determining health outcomes from health care services in both medicine and dentistry [48, 49]. This is particularly pertinent when outcomes are not necessarily associated with mortality but rather have impact on day-to-day living or “quality of life”. In assessing QoL both a generic health-related (by means of the SF-36) and a condition-specific approach (by means of the TOMCP) were employed. The value of employing generic health-related QoL measures is that comparisons can be made with a range of other health outcomes be they prosthetic replacement or other health care interventions. That said, the items of generic health-related QoL measures are such that they are often not sensitive to the subtle changes in QoL associated with orofacial care [50–52]. In terms of physical health (PCS), there was a significant difference over the study period and specifically there was a significant decrease in PCS scores (worse QoL) at 1 week compared to preoperative state. It is not uncommon following surgery or prosthetic placement that sequelae occur (pain, bruising, and swelling) or a need to “adapt” to prosthesis [40]. This would suggest that in timing of evaluating outcomes, consideration should be given for these sequelae to resolve and/or adaptations to occur. Nonetheless, such apparent “negative” changes are important to consider and have implications in informing “informed consent” and patients' decision-making. It was noted that mental health status (MCS) was changed relatively little at different time points of the study, which may indicate that mental health outcomes are unlikely to change following prosthetic placement. This in part may reflect the lack of sensitivity and responsiveness of generic health-related QoL measures to capture such changes.

Condition-specific QoL measures offer a major advantage in that they are “specifically” concerned with the condition or intervention being provided [50]. The Toronto Outcome Measure for Craniofacial Prosthetics (TOMCP) by its very name is concerned with “health outcomes”; these offer specific advantages over simply assessing the effect of a particular condition on QoL and lend themselves more amenable to assess “change” [34]. The TOMCP offers insight into a whole range of QoL (ten domains) covering symptoms/adaptation, physical, psychological, and social well-being. From initial assessment at 1 week postoperative, there was an observed increase in TOMCP scores over time. There was a significant difference in overall/summary TOMCP scores at 1 year compared to 1 week. Furthermore, across the three domains (“Body image”, “Interaction with family/friends/strangers,” and “Mood”) significant changes were observed across the time points observed. Furthermore, for five of the ten domains (“Comfort”, “Aesthetics”, “Body image”, “Interaction with friends/family/strangers,” and “Mood”) there was a significant difference in postoperative domain scores at 3-month, 6-month, or 1-year follow-up compared to 1-week assessment. This highlights the sensitivity of the condition-specific measure to capture changes in QoL. In terms of “comfort” an observed improvement was evident early on, at 3 months compared to 1 week postoperative. This is likely to reflect that initially symptoms are experienced as a result of sequelae of prosthetic placement but that this resolves quickly. It is noted that at 1-year postoperative period, “Comfort” scores were highest. Management of symptoms is of prime concern in all health care and the ability of improvements to be observed early on illustrates the benefits that prosthetic care can bring [53]. Ultimately expected outcomes/health gain from prosthetic care is expected in psychological and social aspects. It was a welcome finding to observe improvements in “mood,” “body image,” and “interactions with family, friends, and strangers”. Impact on social and psychological aspects is referred to as “ultimate” impact aspects as they can result in handicapping effects, which detract from social interaction in personal and working life [48, 49]. The observed “improvements” (increase in domain scores) highlight the responsiveness of the outcome measure to capture such changes, in that not only should there be statistical significant changes, the changes should be in a positive direction—a psychometric attribute of “responsiveness” [51]. It is noted that most improvements were observed in the longer term, at 1-year follow-up, which suggests that when evaluating outcomes from prosthetic placement evaluations should be conducted at least one year postoperatively.

### 4.2. Immediate Prosthesis Connection

Advantages include the following: patients can restore their facial symmetry by immediate restoration of their physical deficiency; psychological benefits to the patients may be expected. Although there was a general trend of improvement in the patients' psychological and QoL outcomes, our sample size was considered too small to draw any definitive conclusion at this stage. It is arguable that an immediate prosthesis connection protocol raises patient's expectation and makes the patient less easily satisfied. Only with factors such as retention and improvement in prosthesis colour can the benefits of immediate connection become more readily realized.

### 4.3. Peri-Implant Soft Tissue

In our 4 HMF cases, the diminished hard and soft tissue in three dimensions presented a challenge in our consideration in their craniofacial rehabilitation [54]. From childhood, when the patient was first examined, every effort was used to improve the craniofacial asymmetry by orthopaedic, orthodontics, orthognathic, or plastic means to increase the soft and hard tissue deficiencies. If the cause of facial asymmetry is congenital such as in HFM, usually the prosthetic auricular reconstruction should be the last stage of a patient's craniofacial rehabilitation process. In this preliminary study, it was observed that the peri-implant soft tissue management played an important role in the final success of the prosthesis. To achieve this, adequate trimming/thinning of the tissue was necessary. Additional soft tissue moulding using the temporary auricular prosthesis during the initial postoperative phase was required and part of the standard procedure to counteract the risk of submerging due to initial inflammatory response. However, our approach needs to be reviewed in light of current bone conduction amplification literature [55, 56] and further observations need to be made for conclusive remarks. There was a tendency of increased risk of submergence of the superior implant. One of the reason can be the close proximity of the temporal region where the temporalis muscle/and part of the scalp are located. The subsequent management using a punch excision of the tissue covering could help to minimize the submergence. This was also recommended by Wolfaardt in using a skin punch or a 3.0 mm diameter (fitted to the implant or abutment) disposable biopsy punch to create holes over the site of the implants [6]. On the other hand, the inferior implant is usually located near the mastoid region where the skin is usually thinner.

### 4.4. Skin Thickness and Abutment Length

In our study, the length of abutment visible (mm) was observed that it was closely related to the level of Holgers scale recorded. The longer the abutment exposed above the skin, the better the outcomes. Although skin thinning had been performed in all patients, some of the soft tissues were still relatively thick for the purpose of good skin conditions (i.e., free from infection and inflammation). Tjellstrom recommended that at least 3 mm of the abutment should be above the skin surface [57] and a skin thickness below 4 mm is desirable [6]. However, recent literature in the bone conduction amplification field reported that preclinical studies demonstrated the potential of a hydroxyapatite-coated abutment coating to enable integration between soft tissue and the abutment. It was claimed that this enhanced dermal adherence and improved soft tissue integration. This enabled a significant reduction in peri-abutment pocket formation and thereby could potentially make safe implant surgery in the temporoparietal regions without soft tissue reduction possible [55]. Also, the soft tissue stability could be enhanced by a special geometric design of an abutment with a pronounced concave shape [56].

### 4.5. Other Soft Tissue Conditions

Delayed hypersensitivity towards metals was suspected in one patient (Case 2) when a persistent skin inflammation was observed [58]. No test was done to confirm this suspicion. However, the inflammation resolved completely when the custom-made abutments were replaced with* Ankylos* sulcus formers (Friadent, Dentsply, Mannheim, Germany), which were made of Titanium alloy.

Part of the difficulties with our study is that we managed in most cases to get a good shape of the ear prosthesis from CAD/CAM; there were still 2 common problems which significantly affect patients' acceptance of the prosthesis: (1) lack of retention, which was closely related to the superior implant abutment exposure, (2) colour mismatch or discoloration of the prosthesis, which was rather common (in 4 out of 6 patients while the remaining 2 did not wear the prosthesis).

### 4.6. Retention (Abutment Attachment Systems)

Although the management of a magnet attachment system is easier in immediate placement by the clinician and during daily connection and removal by the patient, magnets are less retentive. The range of retentive force of* Ankylos* magnet attachment systems (Friadent, Dentsply, Mannheim, Germany) used in our study was about 3 N. This is comparable to the magnet system,* Micro Magnets* (Technovent, Newport, UK) but is much less than that for a bar and clip attachment which is about 16.5 N [59].

### 4.7. Psychology

Body image is a complex concept used to express the mental image of a person's physical self. Our body is the most visible part of our self, occupying a central part of our self-perception. What happens to our body can have an effect on our emotional health and vice versa, since body and mind are closely associated. Any change in our body will invariably cause a temporary or permanent disturbance of our integrity [60]. Whether the patient wears or does not wear his or her ear prosthesis may not be affected by a single factor. It involves a subjective feeling of satisfaction or dissatisfaction affecting acceptance or rejection of the prosthesis. The decision to wear or not to wear reflects their satisfaction or dissatisfaction with the prosthesis. One reason is that they may perceive themselves to be better looking which may be in contrast with any objective measurement such as spectrophotometer for colour, 3D photogrammetry for position, symmetry, size, and shape. Subjective judgment by others including the healthcare professionals may not be the same as the patients' own judgment. Another indication reflecting self-image, self-perception, and self-esteem is by observing their hairstyle. A change of hairstyle that allows revealing of the prosthesis may indicate a positive gain in acceptance and satisfaction (Case 1). In one case (Case 3), it was observed that although there was discoloration in the patient's prosthesis, he adjusted the appearance by applying cosmetics onto his prosthesis. Perhaps the patient might have perceived and treated the prosthesis as if it was a part of his face. During the stage of obtaining patient's consent when the treatment plan is being formulated, it is of paramount importance that the patient understands and has all necessary and relevant information not only for the purpose of making decision but also to generate a realistic and achievable expectation towards treatment. If the patient perceives the goals are fulfilled by the planned treatment, he may be satisfied. In our Hope Scale, the Agency items assess a motivated state to reach desired goals, whereas the Pathways items assess the sense that one will be able to successfully generate a plan to attain them. In the present study, the Agency items reflect the patient's personal and internal state. The Pathway items involve the whole rehabilitation team to generate a plan so that the patient can sense that he or she will be able to successfully generate a plan to attain the items. During the preoperative assessment, the Hope Pathway score (*M* = 22.83, SD = 2.04) was similar to the Hope Agency score (*M* = 22.17, SD = 3.31), comprising an average total Hope score of 45.00 (SD = 4.69). Participants were generally positive, with an average APNIS API score (Positive Attentional Bias) of 15.50, SD = 1.52 and a lower average ANI score (Negative Attentional Bias) of 14.50, SD = 1.38. The difference between the API and ANI subscales was, however, not significant. The preliminary results show that there is a decrease in depressive symptoms after the operation. In addition, positive emotions in terms of happiness level also tend to improve after operation. This suggests that improvement in facial appearance is beneficial to the mood of the patients. There was no significant change in anxiety level in this study. There are two possible explanations to this result. First, the medical condition of the patients may be more a mood issue rather than an anxiety issue. Second, the nonsignificant result may be due to the small number of patients in the analysis. Future study should examine the above two issues. Finally, it should be noted that because of the small number of subjects in this study, the psychological results should be treated with extreme caution. Nevertheless, our present findings can inform research design and hypotheses generation of future studies.

## 5. Conclusion

This preliminary clinical cohort study demonstrates that implant-supported auricular prosthesis manufactured using CAD/CAM technology could induce improvement of the quality of life of patients particularly in the domains of the body image, social interaction, and mood. Our present findings can inform research design and hypotheses generation of future studies.

## Figures and Tables

**Table 1:** Intra-operative complications encountered.

Complications encountered	Superior implant	Inferior implant
	*n* = 6	*n* = 6
During implant placement		
Dura exposure	0	0
Dura perforation	0	0
Bleeding	0	1
During immediate placement of ear prosthesis		
Retention		
Inadequate retention	3	
Unsuccessful bonding	1	
Prosthesis		
poor marginal accuracy	1	
Poor stability	0	
Inadequate function	1	
Asymmetrical/Poor position	0	
Wrong color matching	1	

**Table 2:** Significance of changes in mean PCS scores of the SF-36 over the study period.

Physical component score	Mean	SD	*P*-value*
Pre-operative	56.8	6.5	
1 week	45.9	8.2	0.007*
3 months	53.3	6.6	0.065
6 months	52.7	3.4	0.055
1 year	53.4	3.3	0.156

*P value derived from paired *t*-test comparison with pre-operative scores.

**Table 3:** Significance of changes in mean MCS scores of the SF-36 over the study period.

Mental component score	Mean	SD	*P*-value
Pre-op	45.4	10.6	
1 week	50.0	8.6	0.256
3 months	46.3	9.1	0.783
6 months	47.0	9.6	0.693
1 year	50.3	6.9	0.233

*P value derived from paired *t*-test comparison with pre-operative scores.

**Table 4:** Significance of changes in mean TOMCP-52 scores over the study period.

TOMCP-52	Mean	SD	*P*-value*
1 week	65.3	10.1	
3 months	69.8	11.5	0.245
6 months	70.3	9.9	0.353
1 year	80.7	10.7	0.030*

*P value derived from paired *t*-test comparison with Week 1 scores.

**Table 5:** TOMCP scores over the time points.

Domain score	1 week (mean/SD)	3 months (mean/SD/*P*)	6 months (mean/SD/*P*)	1 year (mean/SD/*P*)
Fit and Retention	62.3/31.3	67.5/14.8/0.682	57.1/19.0/0.714	82.1/14.9/0.370
Comfort	61.7/17.2	78.3/14.7/0.044*	63.3/14.8/0.839	86.1/15.4/0.075
Aesthetics	63.4/10.3	70.4/9.1/0.251	66.7/9.6/0.666	86.6/15.1/0.037*
Maintenance	68.1/20.7	77.8/17.2/0.158	81.9/17.8/0.224	84.7/17.8/0.270
Body image	63.1/12.1	64.3/14.8/0.733	72.2/15.9/0.079	77.4/14.4/0.001*
Leisure	73.3/18.6	74.2/15.8/0.776	78.1/10.8/0.374	77.2/14.2/0.450
Work/school	71.3/21.5	72.2/12.7/0.842	75.0/11.0/0.530	75.9/10.3/0.550
Family/friends/strangers	57.4/15.6	59.3/19.1/0.833	63.9/8.4/0.402	76.9/12.9/0.047*
Mood	60.3/9.2	64.7/11.7/0.304	73.4/12.6/0.039*	81.7/15.5/0.003*
Sexuality	75.0/23.0	73.6/28.1/0.771	79.2/21.6/0.415	79.2/14.7/0.518

*P-values ≤ 0.05 showing significant difference.

*P*: *P*-value derived from paired *t*-test comparison with Week 1 scores.

**Table 6:** Clinical exposure of implants.

Case	Abutment length/mm		Clinical exposure/mm									
	Sup	Inf	Immediate post-op		1-2 weeks post-op		1-month post-op		3-month post-op		6-month post-op	
			Sup	Inf	Sup	Inf	Sup	Inf	Sup	Inf	Sup	Inf
1	6	6	1	0.5	1.25	0.75	2	1	3	3	2	2
2^#^	10	10	4	2	0	0	0	0	NA	NA	0	0
3	4	6	0.5	0.5	1.5	1.5	4.25	3	3	4.8	1.75	3
4^#^	4	6	2	3	0	2.8	0	2.875	0	4.5	0	4
5^#^	4.5	4.5	4.5	4.5	0	1	NA	NA	NA	NA	3	3
6	4.5	4.5	2.5	3	2.5	3	4	3	3.75	3.375	2.25	2

Sup: superior implant; inf: inferior implant;

^#^Patients who refused to wear the ear prosthesis since 3-month postop.

NA: Not Applicable.

**Table 7:** Comparison of the clinical abutment exposure in patients willing and those unwilling to wear the ear prosthesis.

	Immediate post-op		1-2 week post-op		6-month post-op	
	Sup	Inf	Sup	Inf	Sup	Inf
Willing to wear						
Mean exposure (mm)	1.33	1.33	1.75	1.75	2	2.33
Standard deviation	1.04	1.44	0.66	1.15	0.25	0.58
Difference when compares with immediate post-op using pair *t*-test? (*P*-value)			No (0.15)	No (0.15)	No (0.14)	No (0.21)

Unwilling to wear						
Mean exposure (mm)	3.50	3.17	0	1.27	1	2.33
Standard deviation	1.32	1.26	0	1.42	1.73	2.08
Difference when compared with immediate post-op using pair *t*-test? (*P*-value)			Yes (0.02)	No (0.09)	Yes (0.04)	No (0.23)
